# Flexible Polymer–Organic Solar Cells Based on P3HT:PCBM Bulk Heterojunction Active Layer Constructed under Environmental Conditions

**DOI:** 10.3390/molecules26226890

**Published:** 2021-11-15

**Authors:** Georgy Grancharov, Mariya-Desislava Atanasova, Radostina Kalinova, Rositsa Gergova, Georgi Popkirov, Christosko Dikov, Marushka Sendova-Vassileva

**Affiliations:** 1 Institute of Polymers, Bulgarian Academy of Sciences, Akad. G. Bonchev St., Block 103-A, 1113 Sofia, Bulgaria; m.atanasova@polymer.bas.bg (M.-D.A.); kalinova@polymer.bas.bg (R.K.); 2 Central Laboratory of Solar Energy and New Energy Sources, 72 Tzarigradsko Chaussee, 1784 Sofia, Bulgaria; rositsa.gergova@gmail.com (R.G.); popkirov@phys.bas.bg (G.P.); dikov@phys.bas.bg (C.D.); marushka@phys.bas.bg (M.S.-V.)

**Keywords:** polymer–organic solar cells, bulk heterojunction layers, flexible solar cells, current–voltage characteristics, thermal annealing, impedance spectroscopy, device stability

## Abstract

In this study, some crucial parameters were determined of flexible polymer–organic solar cells prepared from an active layer blend of poly(3-hexylthiophene) (P3HT) and the fullerene derivative [6,6]-phenyl-C_61_-butyric acid methyl ester (PCBM) mixed in 1:1 mass ratio and deposited from chlorobenzene solution by spin-coating on poly(ethylene terephthalate) (PET)/ITO substrates. Additionally, the positive effect of an electron transport layer (ETL) prepared from zinc oxide nanoparticles (ZnO np) on flexible photovoltaic elements’ performance and stability was investigated. Test devices with above normal architecture and silver back electrodes deposed by magnetron sputtering were constructed under environmental conditions. They were characterized by current-voltage (I–V) measurements, quantum efficiency, impedance spectroscopy, surface morphology, and time–degradation experiments. The control over morphology of active layer thin film was achieved by post-deposition thermal treatment at temperatures of 110–120 °C, which led to optimization of device morphology and electrical parameters. The impedance spectroscopy results of flexible photovoltaic elements were fitted using two R||CPE circuits in series. Polymer–organic solar cells prepared on plastic substrates showed comparable current–voltage characteristics and structural properties but need further device stability improvement according to traditionally constructed cells on glass substrates.

## 1. Introduction

Polymer–organic solar cells (SCs) have attracted much attention and interest during the last decade. They are an alternative to inorganic SCs, and they offer the achievement of desired optoelectronic properties through chemical tailoring and the possibility of fabrication on flexible substrates via cheap deposition methods, as well as easily affordable large-scale production using low-cost preparation technology. Currently, the best reproducible and cheap construction in the field of polymer–organic photovoltaic elements presents bulk heterojunction solar cells containing an active layer of a widely applied mixture of semiconducting polymer P3HT and organic fullerene derivative PCBM, with an average power conversion efficiency (PCE) up to ~5% [1,2].

Flexible polymer–organic SCs based on different active layer compositions and deposited on plastic substrates have also appeared in the recent years [3,4,5]. They found implementation from flexible wearable solar elements integrated with textiles [6] to stretchable energy harvesting tapes [7] serving as energy sources for supplying self-powered wearable devices. For that purpose, transparent and thermostable polyesters such as PET, poly(ethylene naphthalate) (PEN), poly(ether sulfone) PES, polyimide (PI), and bio-based poly(ethylene furanoate) (PEF) were utilized to replace classical glass substrates [8,9,10]. Additionally, dominant spin-coating process recently introduced a plethora of deposition and printing techniques, such as brush painting [11], inkjet printing [12,13,14], roll-to-roll slot die coating [15,16,17], gravure printing [18], flexographic printing of silver back [19,20], and simultaneous sputtered aluminum top electrodes [21]. Further optimization of the above techniques produced various organic SCs constructed on ultrathin substrates [22,23] and fully printed flexible tandem devices [24].

The necessity to increase the PCE of flexible polymer–organic PVs resulted in extensive investigations of appropriate materials and processes not only connected to the active layer deposition but also comprising the application of transparent interface and metal contact layers. One major improvement in the structure of flexible devices was the replacement of a transparent and conductive indium tin oxide (ITO) layer used as a primary choice for anode contact. However, the disadvantages of ITO, such as its brittle structure and the expensive nature of indium metal resulted in various successful solutions for its exchange with flexible SCs such as silver-grid [25,26], silver nanowires (AgNWs) [27,28,29,30], silver/molybdenum oxide (MoO_3_) layer [31], reduced graphene oxide (rGO) [32,33], aluminum (Al)/chromium (Cr) electrode [34], MoO_3_/Ag/zinc sulfide (ZnS) stack [35], or MoO_3_/metal (Ag or Au)/MoO_3_ trilayer [36]. The next challenge was substitution of the hygroscopic-sensitive and main degradation source in flexible devices—PEDOT:PSS hole transport layer (HTL) that was replaced by vanadium(V) oxide [37]. A great number of studies also investigated the advantages of using inverted rather than standard device structure in flexible polymer–organic SCs. It was found that inverted architecture offers higher efficiency and lower environmental degradation compared with the conventional device structure due to the improved interface stability exchange of unstable PEDOT:PSS with a metal oxide layer [38,39]. In this respect, low-work function metal oxides such as ZnO, titanium oxide (TiOx), etc. were used as an electron transport layer and contributed to the improved performance of inverted photovoltaic elements. Flexible inverted SCs were prepared using ETLs with an annealed ZnO layer [40], a ZnO nanoparticles optimized morphology layer [41], or an Al-doped ZnO or etoxylated polyethylenimine (PEIE)/ZnO bilayer [42]. It was found that the active layer could be structurally characterized on-time via roll-to-roll X-ray scattering [43], and the increase in PCE was obtained by the use of light-trapping, patterned PET substrates [44], and operational stability was analyzed by indoor and outdoor testing [45].

Although the bulk heterojunction P3HT:PCBM solar cells are presently the most widely applied devices, there are still unresolved issues about the relation between preparation method, structure of different layers, and performance of the incorporated materials [46,47]. In this paper the optical, structural, and electrical properties of environmentally prepared flexible SCs based on a P3HT:PCBM active layer were examined as a function of PET substrate behavior, post-deposition thermal annealing, application of ZnO np-based ETL, and device time degradation. The main aim was finding the cheapest and simplest way of preparing flexible polymer–organic photovoltaic elements with reasonable efficiency and stability, similar to those constructed on glass substrates.

## 2. Results and Discussion

**Figure 1:**
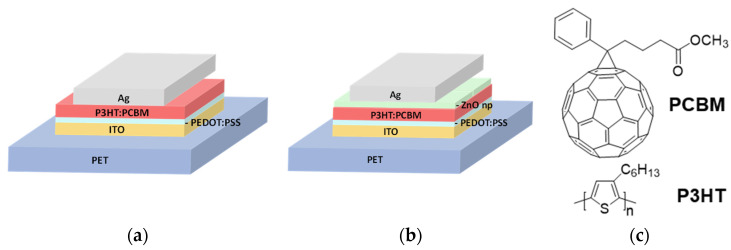
Photovoltaic device architectures: (**a**) without the presence of ETL, (**b**) with the presence of ETL, and (**c**) the chemical structure of active layer ingredients.

Photovoltaic devices were prepared on eight cell ITO-patterned PET substrates under environmental conditions applying a standard configuration (Figure 1). The first variant, ITO-PET/PEDOT:PSS/P3HT:PCBM/Ag, was without the presence of ETL (Figure 1a), and the second variant, ITO-PET/PEDOT:PSS/P3HT:PCBM/ZnO np/Ag, incorporated an ETL based on ZnO nanoparticles (Figure 1b). The materials employed in the active layer were a widely utilized formulation of P3HT:PCBM, as shown in Figure 1c.

**Figure 2:**
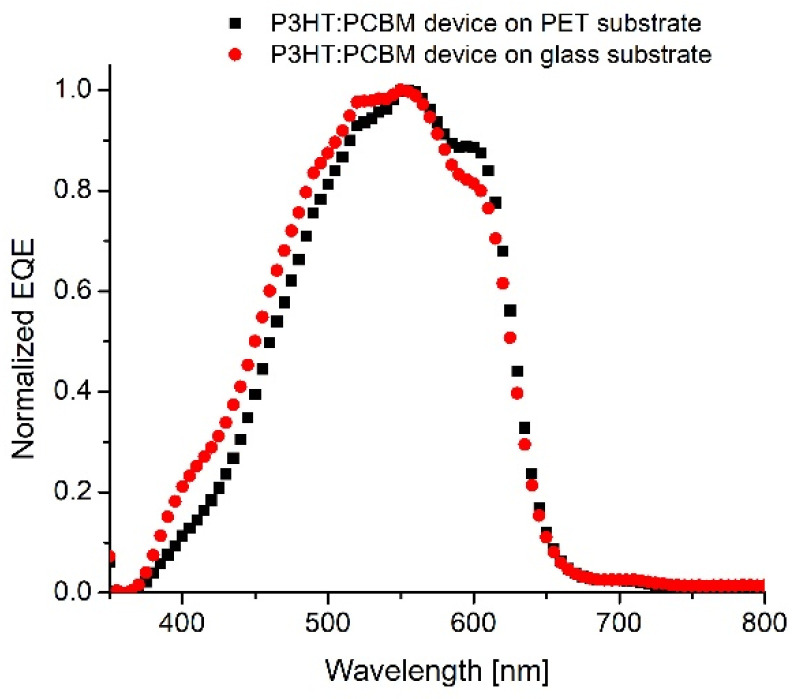
External quantum efficiency spectrum of solar cells.

For comparison, polymer–organic solar cells with the above two types of construction were also fabricated on glass substrates. Looking at the external quantum efficiency spectra in Figure 2, it is well visible that SCs on flexible PET substrates possess similar quantum efficiency in the range of 400–700 nm, similar to those on glass substrates. Consequently, PET substrates can be successfully exchanged with classical glass substrates in photovoltaic devices, as was reported in literature.

### 2.1. Current–Voltage Measurements

**Figure 3:**
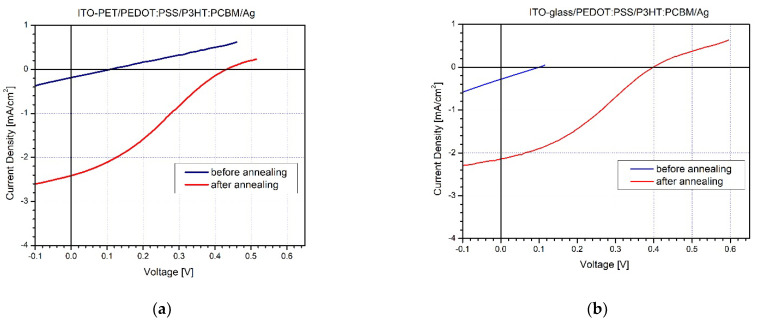
I–V characteristics of P3HT:PCBM based solar cells without ETL constructed in environmental conditions on: (**a**) flexible PET substrates and (**b**) glass substrates.

The I–V characteristics of polymer–organic SCs deposited on flexible PET and rigid glass substrates were measured upon illumination, before and after thermal annealing (Figure 3). The I–V curve of the PET sample before post-annealing (Figure 3a—blue curve) reveals low photocurrent and open circuit voltage as well as very low fill-factor. The main reason for that is the lack of structural orientation of the conjugated polymer P3HT in the bulk heterojunction layers. Such a drawback can be successfully overcome with thermal post-annealing of photovoltaic elements at temperatures of 110–120 °C for 10 min after encapsulation. Significant improvement in the photovoltaic parameters, such as short-circuit current (Isc) and open-circuit voltage (Voc), was obtained after thermal post-annealing of flexible SCs, the values of which increased approximately 5 times (Figure 3a—red curve). Similar results were also observed for the I–V characteristics of photovoltaic elements constructed on classical glass substrates, as shown in Figure 3b (blue and red curve). The performance of the obtained photovoltaic cells without ETL after characterization is presented in Table 1.

Another problem that appeared in the constructed P3HT:PCBM-based SCs with sputtered Ag electrodes was the existence of a characteristic S-shape of the I–V curve, which limits the current flow and lowers the fill-factor. The reason for the S-shape kink has been thoroughly investigated, and it indicates a presence of an energy barrier and a consequent slow charge transfer at one of the metal electrical contacts [48,49,50]. At the moment, the complex circumstances behind the S-shape curve are not fully understood, but we will try to obtain additional information for it by studying the series and shunt resistance values by impedance spectroscopy and by applying atomic force microscopy surface analyses to the SCs layers.

**Figure 4:**
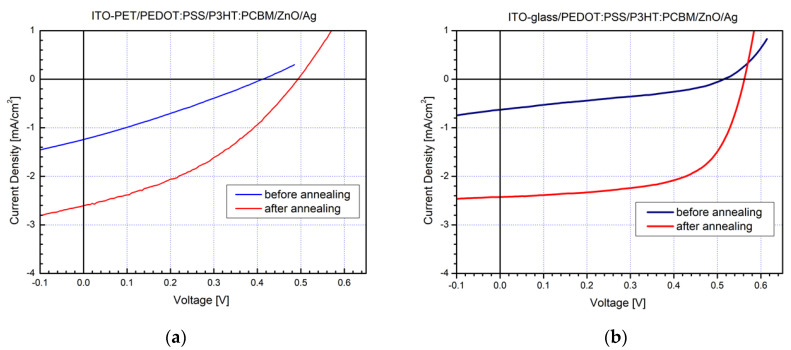
I–V characteristics of P3HT:PCBM-based solar cells with ZnO nanoparticles ETL constructed under environmental conditions on: (**a**) flexible PET substrates and (**b**) glass substrates.

The S-shape kink fully disappeared when ZnO nanoparticles were incorporated as ETL in flexible PET devices. In that case, improved charge transfer between the metal electrode and the inorganic ETL was obviously obtained compared with the previous situation without an ETL application. That was also accompanied by optimized I–V characteristics and higher fill-factors (Figure 4a). Our outcome was also confirmed by photovoltaic elements prepared on classical glass substrates, and their I–V curves are shown in Figure 4b. Presumably, during the magnetron sputtering process, the upper part of the P3HT:PCBM active layer was impaired in the absence of ETL, causing that S-shape kink, and alternatively, it was protected in the presence of ZnO ETL imparting the improved I–V curves and fill-factor.

The photovoltaic values of the prepared SCs with ETL after characterization are presented in Table 1.

### 2.2. Impedance Spectroscopy Measurements

Impedance spectroscopy measurements were applied extensively to solar cells for characterization of important parameters, such as carrier lifetime and electron density-of-states [51], mobility [52], and density and energetic position of defect states in the absorbing layer [53]. Impedance spectra can be obtained in a wide frequency range, at different bias voltage, and with or without illumination by applying proper calculation models [54].

**Figure 5:**
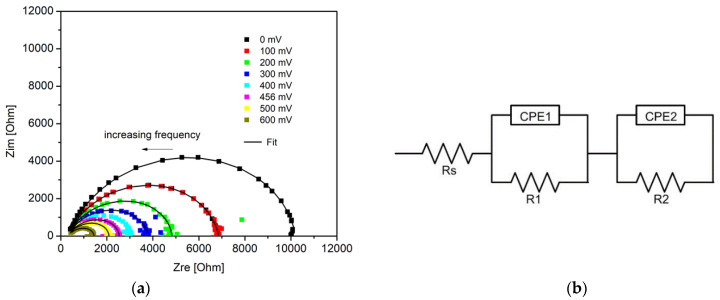
Impedance spectra in dependence of applied forward voltage (**a**) and equivalent circuit used for modeling (**b**) of flexible P3HT:PCBM-based solar cells constructed on PET substrates.

Figure 5a presents the impedance spectra of flexible solar cells with a ZnO electron transport layer in dependence on applied forward voltage through the working area of the solar cell. This diagram appears as a single depressed semicircle in the complex impedance plane, but the spectra could not be fitted with the simple R||CPE equivalent circuit model. A constant phase element (CPE) is commonly used to represent a distributed time constant. We obtained the best fits using a model consisting of two R||CPE circuits connected in series, which is presented in Figure 5b. Rs represents the series resistance, which consists of the electrodes and connecting wires resistances. Normally, under short circuit conditions (0 V), all the charges are extracted from the device, and a single R||CPE circuit describes the impedance spectra of the organic solar cells [55]. In our case, the presence of an additional time constant under conditions close to short circuit probably points to a charge accumulation at one of the interfaces, which leads to a reduction in charge collection efficiency. Impedance spectroscopy cannot discern which interface is responsible for the inefficient charge collection, but we suppose that R1||CPE1 corresponds to the problem interface and R2||CPE2 corresponds to the bulk heterojunction of the SCs. This additional process observed in the impedance spectra contributes to the high series resistance evident in the I–V characteristics. The impedance spectra at 0 V for devices on PET substrates with and without ZnO ETL were comparable, with only differences in the parallel resistances (R2), which under these conditions represent the shunt resistance of the device. The shunt resistance of the devices with ZnO ETL (401 Ω cm^2^) was larger than the shunt resistance of the device without ETL (333 Ω cm^2^). The results from the impedance spectra under conditions close to short circuit were in support of the data obtained from the I–V curves. These results indicate that the insertion of ZnO improves series resistance, but it can also lead to an improvement in the shunt resistance parameter.

### 2.3. Device Stability Characterization

**Figure 6:**
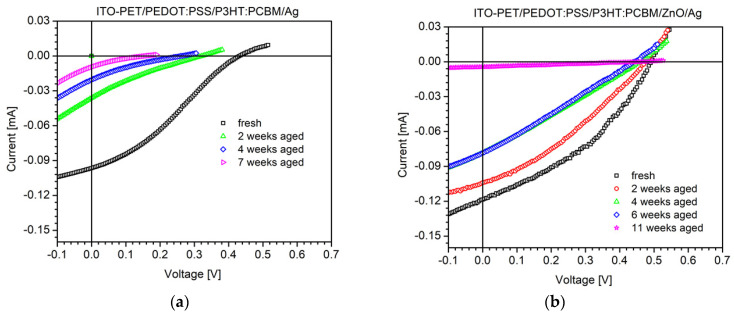
Change in I–V characteristics of P3HT:PCBM-based solar cells on flexible PET substrates constructed under environmental conditions after degradation: (**a**) without presence of ZnO nanoparticles ETL and (**b**) in the presence of ZnO nanoparticles ETL.

Device stability of polymer–organic SCs was analyzed under shelf-life stability tests for different time intervals. Encapsulated flexible photovoltaic devices without ETL were stable for 7 weeks, whereas those with ETL of ZnO nanoparticles were stable for 11 weeks. The change in the I–V characteristics with the time of both types of photovoltaic elements is shown in Figure 6.

It was found that faster degradation appeared in the first 24 h of shelf-life test studies for both types of photovoltaic elements. After that the degradation process continued at a much slower speed. This was also accompanied by a significant decrease in I sc and Voc associated with the penetration of oxygen and water into the photovoltaic elements due to possible improper encapsulation by PET laminate sheets. Flexible SCs without ETL (Figure 6a) degraded at a much faster rate compared with flexible devices containing ETL (Figure 6b), which is another advantage of the application of such a layer. Regardless, finding considerable improvement of the encapsulation technique for flexible photoelements is needed before more reliable and stable devices can be prepared, such as those prepared on the similar glass substrates that started to degrade only after 40 weeks of storage [56].

### 2.4. AFM Surface Characterization

**Figure 7:**
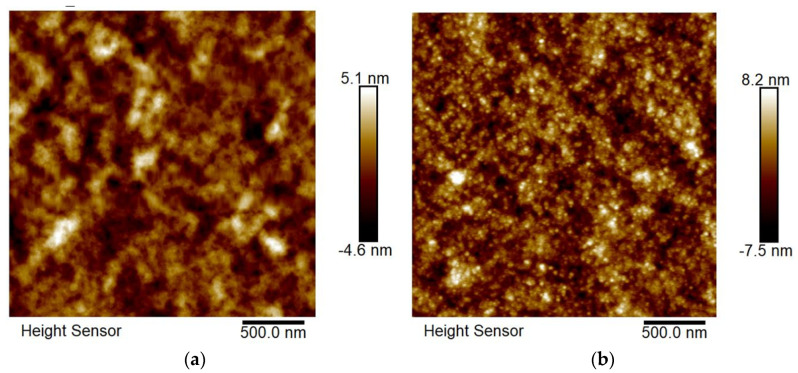
AFM tapping mode height images of: (**a**) P3HT:PCBM active film and (**b**) ZnO interface layer of flexible polymer–organic solar cells.

The influence of surface morphology on BHJ P3HT:PCBM active film and the ZnO thin interface layer of flexible polymer–organic solar cells was revealed by AFM investigations. Both of the layers were annealed at temperatures of 110–120 °C, and AFM tapping mode height images were performed (Figure 7).

Isolated domains at about 120 nm, an interpenetrating network, and the presence of nanofibrillar domains are typical features of an active BHJ layer on the image of a P3HT:PCBM blend (Figure 7a). Quite smaller domains at about 30 nm and a continuous interpenetrating network were observed on the image of the ZnO electron transport layer (Figure 7b). The latter type of morphology is favorable for the efficient charge transport between the metal electrode and the ETL of photovoltaic devices. Additionally, that observation is connected to the higher fill-factor and improved J–V characteristics of flexible SCs possessing a ZnO interface layer.

## 3. Materials and Methods

### 3.1. Materials

PET/ITO-patterned substrates (VisionTek Systems, Chester, UK), glass/ITO-patterned substrates (Ossila, Sheffield, UK), regioregular-conjugated polymer P3HT-Sepiolid P200 (BASF, Germany), fullerene derivative PCBM ([60]PCBM from Solenne BV, Groningen, The Netherlands)), PEDOT:PSS (PVP AI 4083 HC Clevious, Germany), ZnO nanoparticles ink suspension with particles size 10–15 nm (SigmaAldrich–Merck, Burlington, MA, USA). All of the solvents—chlorobenzene, isopropanol, acetone, and methanol—were supplied by SigmaAldrich–Merck, USA and were used as received.

### 3.2. Device Fabrication

Polymer–organic active layers of P3HT and the fullerene derivative PCBM mixed in 1:1 mass ratio were deposited by spin-coating from a chlorobenzene solution with a concentration of 12 mg/mL prepared by stirring the components for 48 h at 50 °C. PET and glass ITO-patterned substrates containing eight separate pixels with an active area of 4 mm^2^ were cleaned in ultrasonic bath by acetone, isopropanol, and methanol for 10 min and were dried under nitrogen flow. The dry substrates were treated additionally by UV–ozone for 15 min. The PEDOT:PSS layer was then spin-coated at 1750 rpm and subsequently thermally treated at 120 °C for 15 min. After that, deposition of the active films was performed at 400 rpm under environmental conditions. The ETL of ZnO nanoparticles from isopropanol solution was spin-coated at 2000 rpm on top of the active layer. The Ag back electrode contacts were deposited by magnetron sputtering. The photovoltaic elements were encapsulated by PET and glass laminate sheets for flexible PET and rigid glass substrates, respectively. Finally, all of the devices were subjected to post-deposition thermal annealing at 110–120 °C for 10 min in an environmental atmosphere.

### 3.3. Characterization

The I–V curves of solar cells were measured with a Keithley 2400 source meter (USA) under illumination and in the dark. For that purpose, a halogen lamp was used for the measurements under illumination providing 100 mW/cm^2^ light intensity, and a Si photodiode was applied as a reference. The quantum efficiency analyses were performed by a computer controlled system including a Digikrom 240 monochromator (USA), a lock-in amplifier, a chopper, a halogen lamp, and a referent Si photodiode. The impedance spectra were taken with a fast Fourier transform (FFT) impedance spectrometer (Germany) in the time domain employing a multi-sine perturbation signal with an amplitude of 25 mV. The spectra were characterized in the frequency range from 1 mHz to 100 kHz under standard illumination. EIS Spectrum Analyzer software (Belarus)was used for fitting the impedance spectra. AFM images were collected using a Bruker Dimension Icon atomic force microscope (USA) that was operated in tapping mode using silicon cantilevers with resonance frequencies of approximately 300 kHz and spring constant of 40 N/m. Shelf-life stability tests of the photovoltaic elements were performed in compliance with the ISOS-D1 standard testing protocol [57]. The devices were kept in ambient temperature (23 ± 4 °C), in ambient relative humidity (50 ± 15%), in the dark, and current–voltage measurements were taken at regular time intervals.

## 4. Conclusions

Polymer–organic solar cells based on a bulk heterojunction P3HT:PCBM active layer were successfully constructed on plastic PET substrates under environmental conditions. It was found that the mass ratio of donor (P3HT)/acceptor (PCBM) = 1:1 in the active layer, with chlorobenzene as a solvent, an ITO anode, PEDOT:PSS as hole transport layer, ZnO nanoparticles as electron transport layer, and sputtered Ag cathode were the most appropriate circumstances for fabrication of cheap and simple flexible SCs. They showed current–voltage characteristics and power conversion efficiency similar to the photovoltaic cells constructed on classic glass substrates. Post-deposition thermal annealing at 110–120 °C increased by 5 times the photovoltaic parameters, such as Isc, Voc, and PCE of flexible SCs. The incorporation of ZnO nanoparticles-based ETL that is spin-coated on the top of an active layer caused more pronounced impact on the FF value and optimized the I–V characteristics, eliminating their S-shape, a feature typical for devices without ETL. The ZnO layer also produced a favorable effect on the device time stability of the flexible photovoltaic elements, whereas their impedance spectroscopy model was fitted with two R||CPE circuits in series. Further improvement in encapsulation of flexible solar cells, followed by reduced time degradation is necessary to obtain comparable efficiency and stability to traditionally constructed photovoltaic cells on glass substrates.

## Figures and Tables

**Table 1:** Photovoltaic parameters of solar cells.

Type of Photoelement	Voc, V	Jsc, mA/cm^2^	FF, %	PCE, %
ITO-PET/HTL/active layer/Ag	0.434	2.74	27	0.34
ITO-glass/HTL/active layer/Ag	0.451	2.52	36	0.42
ITO-PET/HTL/active layer/ETL/Ag	0.496	2.60	38	0.49
ITO-glass/HTL/active layer/ETL/Ag	0.561	2.49	61	0.86

## Data Availability

All data and analyses are available in the manuscript.

## Sample Availability

Samples of the compounds and solar cells are available from the authors.
