# Correction: Grancharov et al. Flexible Polymer–Organic Solar Cells Based on P3HT:PCBM Bulk Heterojunction Active Layer Constructed under Environmental Conditions. *Molecules* 2021, *26*, 6890

**DOI:** 10.3390/molecules28114556

**Published:** 2023-06-05

**Authors:** Georgy Grancharov, Mariya-Desislava Atanasova, Radostina Kalinova, Rositsa Gergova, Georgi Popkirov, Christosko Dikov, Marushka Sendova-Vassileva

**Affiliations:** 1 Institute of Polymers, Bulgarian Academy of Sciences, Akad. G. Bonchev St., Block 103-A, 1113 Sofia, Bulgaria; m.atanasova@polymer.bas.bg (M.-D.A.); kalinova@polymer.bas.bg (R.K.); 2 Central Laboratory of Solar Energy and New Energy Sources, 72 Tzarigradsko Chaussee, 1784 Sofia, Bulgaria; rositsa.gergova@gmail.com (R.G.); popkirov@phys.bas.bg (G.P.); dikov@phys.bas.bg (C.D.); marushka@phys.bas.bg (M.S.-V.)

The authors would like to correct a mistake in Figure 3 as published in the original publication [1].

The corrected Figure 3 appears below.

**Figure 3:**
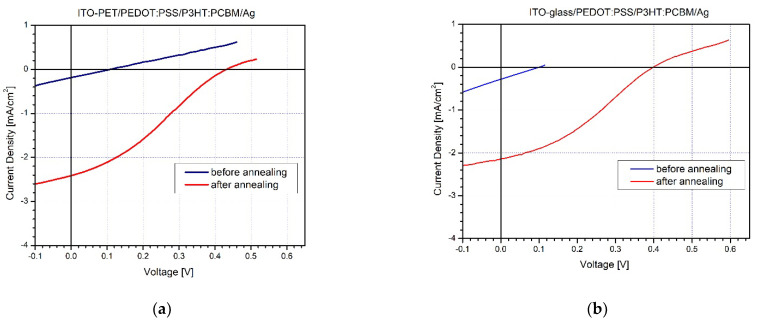
I–V characteristics of P3HT:PCBM based solar cells without ETL constructed in environmental conditions on: (**a**) flexible PET substrates and (**b**) glass substrates.

The authors apologize for any inconvenience caused and state that the scientific conclusions are unaffected. The original publication has also been updated.
